# Management of Pediatric Septic Shock and Acute Respiratory Distress Syndrome in Thailand: A Survey of Pediatricians

**DOI:** 10.3389/fped.2021.792524

**Published:** 2022-01-12

**Authors:** Pasita Puttiteerachot, Nattachai Anantasit, Chanapai Chaiyakulsil, Jarin Vaewpanich, Rojjanee Lertburian, Marut Chantra

**Affiliations:** ^1^Division of Pediatric Critical Care, Department of Pediatric, Faculty of Medicine, Ramathibodi Hospital, Mahidol University, Bangkok, Thailand; ^2^Division of Pediatric Critical Care, Department of Pediatrics, Faculty of Medicine, Thammasat University Hospital, Thammasat University, Bangkok, Thailand

**Keywords:** septic shock, acute respiratory distress syndrome, survey, pediatrician, Thailand

## Abstract

**Introduction:** Pediatric septic shock and acute respiratory distress syndrome (pARDS) are major causes of morbidity and mortality in pediatric intensive care units (PICUs). While standardized guidelines for sepsis and pARDS are published regularly, their implementation and adherence to guidelines are different in resource-rich and resource-limited countries. The purpose of this study was to conduct a survey to ascertain variation in current clinician-reported practice in pediatric septic shock and acute respiratory distress syndrome, and the clinician skills in a variety of hospital settings throughout Thailand.

**Methods:** We conducted an electronic survey in pediatricians throughout the country between August 2020 and February 2021 using multiple choice questions and clinical case scenarios based on the 2017 American College of Critical Care Medicine's Consensus guideline for pediatric and neonatal septic shock and the 2015 Pediatric Acute Lung Injury Consensus Conference.

**Results:** The survey elicited responses from 255 pediatricians (125 general pediatricians, 38 pulmonologists, 27 cardiologists, 32 intensivists, and 33 other subspecialists), with 54.5% of the respondents having <5 years of PICU experience. Among the six sepsis scenarios, 72.5 and 78.4% of the respondents had good adherence to the guidelines for managing fluid refractory shock and sedation for intubation, respectively. The ICU physicians reported greater adherence during more complex shock. In ARDS scenarios, 80.8% of the respondents reported having difficulty diagnosing ARDS mimic conditions and used lesser PEEP than the recommendation. Acceptance of permissive hypercapnia and mild hypoxemia was accepted by 62.4 and 49.4% of respondents, respectively. The ICU physicians preferred decremental PEEP titration, whereas general pediatricians preferred incremental PEEP titration.

**Conclusion:** This survey variation could be the result of resource constraints, knowledge gaps, or ambiguous guidelines. Understanding the perspective and rationale for variation in pediatricians' practices is critical for successful guideline implementation.

## Introduction

Pediatric septic shock and pediatric acute respiratory distress syndrome (pARDS) are the leading causes of morbidity and mortality in pediatric intensive care units (PICUs) worldwide. Mortality rates range from 4 to 50% in sepsis (1–5) and 10 to 33% in pARDS (6), depending on the severity of the illness, risk factors, and geographic location. A recent multicenter Asian study found that pediatric septic shock had a mortality rate of 19.2% (7), while pARDS had a mortality rate of 30.3% (8). The American College of Critical Care Medicine (ACCM) (9) and the Pediatric Acute Lung Injury Consensus conference group (PALLIC) (10) had regularly published guidelines and recommendations for sepsis and pARDS to standardize patient care and improve outcomes. Adherence to these guidelines had been shown to reduce the mortality in pediatric septic shock from 38 to 8%; however, only 30% of the resuscitation practice adheres to standards (11). The lung-protective ventilation strategy such that of low tidal volume ventilation had been shown to reduce mortality in patients with ARDS (12). However, in an observational study, 25% of pediatric patients were ventilated with >10 ml/kg of expiratory tidal volumes (13). The guidelines were implemented and adhered throughout the world. Thailand, one of the developing country in Southeast Asia, is divided into 76 provinces and a capital city. Our country's population is predicted to be 66 million, with over 13 million children. Due to the shortage of pediatric ICU physicians in our country, other specialists and general pediatricians manage the majority of critical care in PICU, which might result in a greater variation in the management and less adherence to guidelines.

Thus, we decided to conduct this self-reported survey to describe pediatricians' knowledge in the management of pediatric septic shock and pARDS. The objectives of this study were to evaluate pediatricians' knowledge compared to guidelines and assess practice variation among ICU and non-ICU physicians as well as the capability of physician skills across various types of hospitals.

## Methods

### Study Design

We developed a cross-sectional, self-administered survey to assess pediatricians' stated septic shock and pARDS practice patterns. Pediatricians with at least 1 year of experience working in pediatric intensive care units were eligible, whose worked in the tertiary care hospitals or higher. Currently, there are approximately 51 PICUs, comprises of 31 tertiary care hospitals, twelve university hospitals, and eight private hospitals. Over the last three decades, pediatric pulmonologists, pediatric cardiologists, and some general pediatricians have been tasked with the responsibility of caring for critically ill children in the PICU. For example, they were able to perform tracheal intubation and manage the ventilator settings, as well as administer fluid resuscitation and inotropic therapy due to the unavailability of pediatric intensivists. There were no respiratory therapists, clinical pharmacists, or nutritionists in our country. However, critically ill children now faced more challenges than in the past. Since 2015, the Thai Society of Pediatric Respiratory and Critical Care Medicine has established a pediatric critical care fellowship training program (TPRC). At the time of writing, Thailand has 32 pediatric intensivists, with the majority of them based in Bangkok (capital city of Thailand). The TPRC hosts two academic conferences, six interhospital critical care conferences, and 2–3 ventilatory management workshops each year to ensure that both ICU and non-ICU physicians have adequate critical care knowledge. In addition, in 2018, the TPRC issued the evidence-based guideline for the management of Thai pediatric sepsis and septic shock. In our country, pediatric intensivists, pulmonologists, and cardiologists were the majority of pediatricians who cared for critically ill children in the PICU and were considered to be the ICU physicians. Nevertheless, in some hospitals with PICU, there were no available ICU physicians, therefore all the critically ill children in those hospitals would be taken care of by the general pediatricians or other pediatric subspecialties. Thus, in this study, we divided the enrolled pediatricians into two categories: the ICU physicians (pediatric intensivists, pulmonologists, and cardiologists) and the non-ICU physicians.

### Survey Development

The survey questionnaire was developed in accordance with the 2014 American College of Critical Care Medicine consensus guideline for pediatric and neonatal septic shock (9) and the 2015 Pediatric acute lung injury consensus conference (10), to assess current practices and knowledge among Thai pediatricians. The authors drafted the questionnaire following a thorough review of the literature and had it reviewed by four pediatric intensivists for clarity, consistency, objectivity, content validity, and completion time. The questionnaire was modified and finalized based on the feedback following a pilot survey of 15 pediatricians from our center who were not the participants of this study.

The final survey included three domains: (I) demographic data of physicians and hospitals, (II) clinical skills, and (III) six clinical case scenarios for sepsis and six clinical case scenarios for pARDS, each of which assessed a different component of the guidelines for the diagnosis and management. The questionnaire for each clinical scenario included questions regarding fluid-refractory shock, sedation for intubation, catecholamine-resistant shock, normotensive shock with increased systemic vascular resistance (SVR), hypotensive shock with decreased SVR, and refractory vasoplegic shock, shown in Table 1. The questionnaires for pARDS included questions about diagnosis, ventilator strategies in mild ARDS, optimal positive end-expiratory pressure (PEEP) in severe ARDS, lung protective strategies, PEEP titration, and recruitment maneuver, shown in Table 2. Tables 1, 2 were the case scenarios that represent in each objective of pediatric septic shock and pARDS. To avoid misinterpretation, all advanced hemodynamic parameter reference ranges were clearly stated. Each scenario had multiple-choice answers, and adequate knowledge was defined as the appropriate answer in accordance with the ACCM and PALICC guideline. For example, the first case scenario with a fluid-refractory shock patient, the proper response would be norepinephrine or epinephrine infusion. This study was approved by the institutional review board (IRB). Online informed consent was obtained prior to enrollment. Respondents were voluntary and anonymous.

**Table 1:** Description of the six scenarios of pediatric septic shock.

**Scenario 1:** A 2-year-old boy, known case of acute lymphoblastic leukemia, who received an induction phase of chemotherapy, presents with septic shock. He receives 40 ml/kg of isotonic crystalloid solution and appropriate antibiotic. Body temperature 39.5°C, HR 170/min, RR 30/min, capillary refill 2 sec, BP 80/30 mmHg., SpO_2_ 98% (O_2_ cannula 2 LPM), warm extremities, Lungs: fine crepitation both lungs, mild distress, mild chest retraction. Initial arterial lactate 4 mmol/L. Which of the following is the next step of appropriate management?
**Scenario 2:** As information above, he develops respiratory failure and requires intubation. Which of the following is the sedation of choice?
**Scenario 3:** As information above, his HR is 150/min, BP 80/55 mmHg while receiving 0.2 mcg/kg/min of norepinephrine and 0.1 mcg/kg/min of epinephrine. His lactate and ScvO_2_ are 5 mmol/L and 75%, respectively. Non-invasive monitoring shows adequate preload, normal cardiac index, and LVEF of 60%. Which of the following is the next management?
**Scenario 4:** A previously healthy 8-year-old girl was admitted to the PICU for septic shock. She received a total of 60 ml/kg of fluid resuscitation through an internal jugular venous catheter and appropriate antibiotics. Epinephrine was titrated up to 0.2 mcg/kg/min. At PICU: Body temperature 39°C, HR 170/min, RR 40/min, capillary refill is 4 s, ABP 100/70 mmHg, Cold extremities, good peripheral pulse. Hb 12 g/dL, ScvO_2_ 60%, Lactate 5 mmol/L. Ultrasound shows adequate preload without pericardial nor pleural effusion. Which of the following is the appropriate inotrope/vasopressor?
**Scenario 5:** A 6-year-old boy, BW 20 kg, presents with severe pyelonephritis and septic shock. He received a total of 60 ml/kg of fluid and epinephrine was titrated to 0.3 mcg/kg/min. Body temperature 39.5°C, HR 170/min, RR 45/min, capillary refill 5 s, ABP 48/32 (39) mmHg, cold extremities, weak central pulse. CVP 13 cmH_2_O, lactate 10 mmol/L, ScvO_2_ 60%, Hb 11 g/dL. Urine output was 0.2 cc/kg/hr. Bedside ultrasound reveals LVEF of 55%, distended IVC, and diffused B-line from lung ultrasound. Non-invasive monitoring shows CI 8.3L/min/m^2^, SVRI 507 dyns/sq.mm/m^2^ (normal range 1000–2000 dyns/sq.mm/m^2^). Which of the following is the most appropriate management?
**Scenario 6:** A 1-year-old boy, BW 8 kg, known case of biliary atresia presents with spontaneous bacterial peritonitis and septic shock. He received a total of 60 ml/kg of NSS and norepinephrine was titrated to 0.2 mcg/kg/min. At PICU: Body temperature 39°C, HR 160/min, RR 50/min, capillary refill is 1 s, ABP 70/35 (45) mmHg, warm extremities, and bounding peripheral pulses. Non-invasive hemodynamic monitoring (USCOM) shows CI 7.9 L/min/m2, SVRI 717 dyns/sq.mm/m2 (normal range 800–1200 dyns/sq.mm/m2), SV 15 ml (normal range 1.5-2.25 ml/kg). What is your next step of management?

**Table 2:** Description of the six scenarios of pediatric acute respiratory distress syndrome.

**Scenario 1:** A 1-month-old boy, previously healthy with no postnatal complication, presents with 3 days of URI symptoms and later develops respiratory failure. He is intubated and ventilated in a pressure control mode PC 10 PEEP 5 FiO_2_ 0.6 (SpO_2_ 85%). ABG shows pH 7.38, PCO_2_ 42 mmHg, PaO_2_ 50 mmHg, Oxygenation index = 8, ScvO_2_ 90%. Physical examination reveals fine crepitation both lungs without cardiac murmur. CXR shows pulmonary congestion. Do you diagnose this patient with pediatric ARDS?
**Scenario 2:** A 1-year-old boy presents with pneumonia and respiratory failure. He is on high flow nasal cannula with FiO_2_ of 0.5. ABG shows pH 7.35, PCO_2_ 38 mmHg, PO_2_ 105 mmHg, HCO_3_ 19 mmol/L. He is diagnosed with pediatric ARDS. He is intubated and sedated. Which of the following is the initial ventilator setting?
**Scenario 3:** An 11-year-old girl, known case SLE, was admitted to the PICU with pulmonary hemorrhage. She is intubated and ventilated in a pressure control mode PC 16 above PEEP 6 FiO_2_ 0.6 (SpO_2_ 88%) Pmean 14. ABG shows pH 7.35, PCO_2_ 35 mmHg, PO_2_ 50 mmHg, HCO_3_ 19 mmol/L. Which of the following is your management on ventilator setting?
**Scenario 4:** Which of the following are the lung protective strategies for severe pARDS?
**Scenario 5:** A 5-year-old boy visits a general hospital with a diagnosis of community-acquired pneumonia. He was intubated and ventilated with a pressure control mode, pressure above PEEP 20, PEEP 5, RR 30 (Pmean 16), TV 5 ml/kg. His SpO_2_ is 85%, FiO_2_ was increased to 1.0 to maintain SpO2 90–92%. His hemodynamic parameters are stable. Initial ABG shows: pH 7.294 PaO_2_ 60 mmHg (FiO_2_ 1.0) -> oxygenation index 26, PaCO_2_ 34.5 mmHg HCO_3_ 16.7 mmol/L. His diagnosis was pediatric ARDS. He was referred to your hospital. Which of the following is the next step on ventilator management?
**Scenario 6:** Do you plan to do the lung recruitment maneuver in moderate to severe ARDS patient? What is your method of lung recruitment maneuver?

### Distribution of Surveys

Our country had 31 tertiary-level hospitals, 12 university-level hospitals, and eight private hospitals with pediatric intensive care units. The survey was distributed via electronic mail to all registered general pediatricians, pediatric intensivists, pulmonologists, and cardiologists working in these hospitals, and was followed up with 2 monthly email reminders. Participants provided their consent and the information was kept confidential. Participants were asked to electronically sign the informed consent before answering the survey. Data were collected automatically using an electronic survey engine (Google Form). After we received responses from the participants, we rechecked that the responses were not duplicates. Initially, we received a low response rate. Therefore, we attempted to announce on several national academic conferences and social media platforms such as Line and Facebook during the study period. The survey was opened between August 2020 and February 2021.

### Statistical Analyses

Statistical analyses were performed using SPSS software (version 23, SPSS, Inc., Chicago, Illinois, USA). Descriptive variables were analyzed as absolute frequencies, percentages, means, and standard deviations. Comparisons of categorical variables across different groups were assessed using a Chi-square test or Fisher's exact test and we used a Student *t*-test for continuous variables. A two-tailed *p* <0.05 was considered statistically significant.

## Results

A total of 255 pediatricians responded to the survey. The demographic and baseline characteristics were illustrated in Table 3. The majority (74.5%) were females, and general pediatricians (49%). Almost all responders had spent <10 years in the PICU (86.7%). There were 118 (46.3), 97 (38), 86 (33.7), and 197 (77.2%) respondents who have experience in using video laryngoscope, laryngeal mask airway, non-invasive hemodynamic monitoring, and ultrasound-guided vascular access, respectively. Furthermore, there were only 65 (25.5%) respondents who have experience in the initiation of an extracorporeal membrane oxygenator.

**Table 3:** Demographic data and baseline characteristics of participants.

**Characteristics**	**Participants (*n* = 255)**
Female, *n* (%)	190 (74.5)
Age, mean (SD)	35.7 (6.9)
**Pediatric subspecialties**, ***n*** **(%)**	
General pediatrician	125 (49)
Pulmonologist	38 (14.9)
Cardiologist	27 (10.6)
Intensivist	32 (12.5)
Other subspecialists	33 (12.9)
**Years of PICU experience**, ***n*** **(%)**	
<5	139 (54.5)
5–10	72 (28.2)
>10	44 (17.3)
**Workplace**, ***n*** **(%)**	
Tertiary hospital	139 (54.5)
Medical school	78 (31.6)
Private hospital	38 (14.9)
**Type of PICU**, ***n*** **(%)**	
Mixed PICU	158 (61.9)
Medical PICU	82 (32.2)
Adult mixed ICU	11 (4.3)
Cardiac PICU	4 (1.6)

*PICU, pediatric intensive care unit*.

### Practices for Sepsis Management

**Figure 1:**
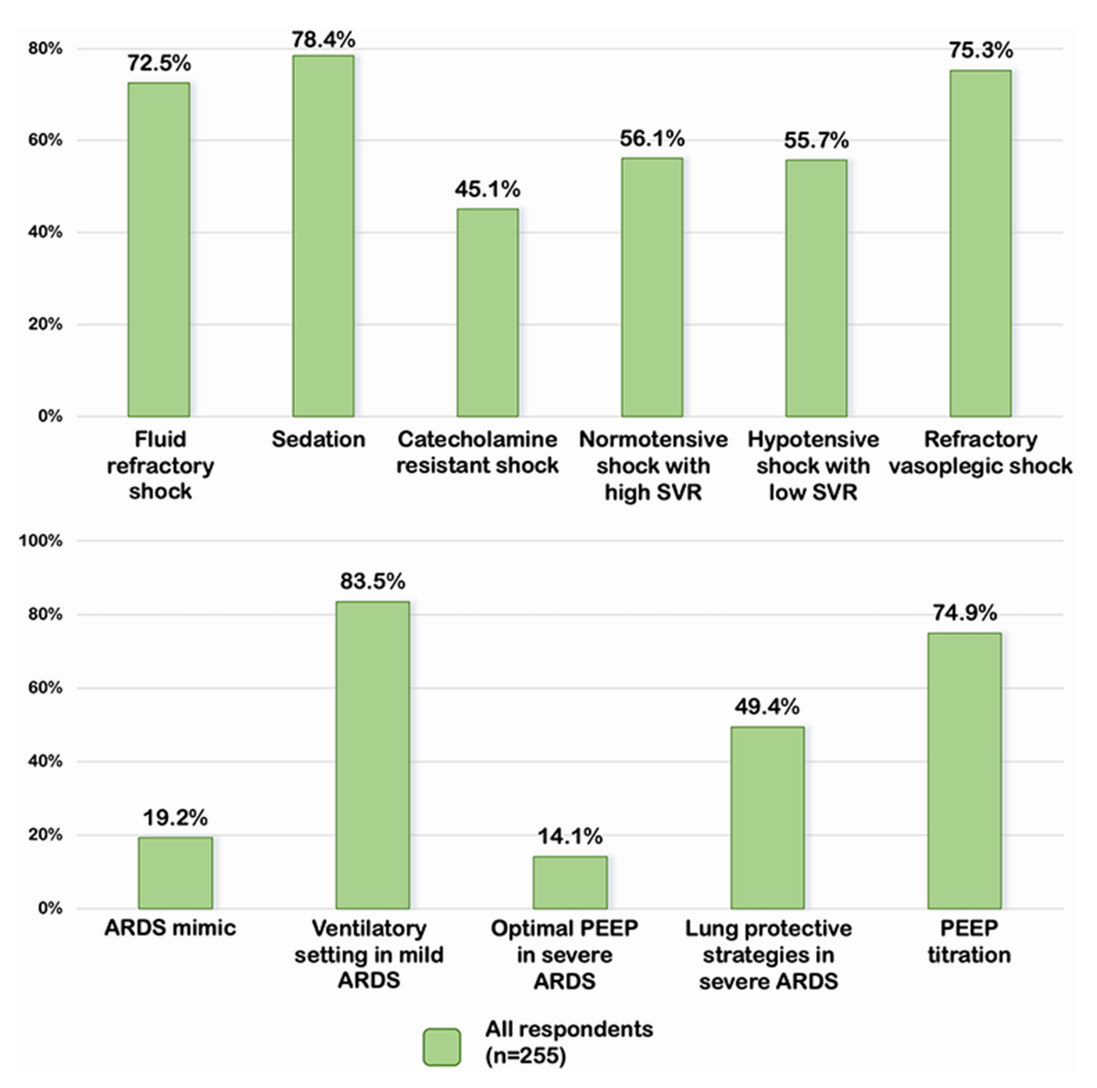
Percentage of appropriate answers on septic shock and pARDS in all respondents. SVR, systemic vascular resistance; PEEP, positive end expiratory pressure; ARDS, acute respiratory distress syndrome.

Overall, 185 (72.5), 200 (78.4), 115 (45.1), 143 (56.1), 142 (55.7), and 192 (75.3%) of the respondents demonstrated adequate knowledge of pediatric septic shock management in each clinical scenario (Figure 1). Almost three-quarters of the respondents indicated that norepinephrine should be the first inotrope/vasopressor of choice in fluid-refractory shock with wide pulse pressure. The most frequently prescribed sedative medications for intubation were a combination of fentanyl and midazolam (49.8%), while 21.6 % of respondents chose etomidate in combination with other sedative medications. Approximately 45.1% of the respondents prescribed corticosteroids in patients with catecholamine-resistant shock, while only 4.7% conducted random cortisol levels prior to initiating corticosteroids. Around half of the respondents (46.3% for milrinone and 9.8% for dobutamine) prescribed vasodilator medications to patients who were in normotensive shock with high SVR. Over half of the respondents would add norepinephrine in hypotensive shock with low SVR patients, while 12.9 and 7.1%, respectively, would increase epinephrine and dopamine to high doses. In refractory vasoplegic shock, the majority of respondents (49.8%) would increase norepinephrine and 25.5% would add terlipressin as the vasopressor.

### Practices for PARDS Management

A total of 206 (80.8%) of respondents misdiagnosed the patient in scenario 1 with pARDS rather than total anomalous pulmonary venous return with obstruction which was the ARDS mimic conditions (Figure 1). The majority of the respondents followed the Pediatric Acute Lung Injury Consensus conference group (PALLIC), which preferred using the pressure-controlled mode, 5–8 ml/kg of tidal volume, 5–7 cmH_2_O of positive end-expiratory pressure (PEEP), and limited the plateau pressure to less than or equal to 28 cmH_2_O in mild pARDS patients. Only 14.1% of respondents reported using an adequate PEEP of 10–15 cmH_2_O, while the majority reported using PEEP less than the recommendation in patients with severe pARDS. The permissive hypercapnia with mild acidosis (pH 7.15–7.30) was accepted as the optimal strategy for 50.2% of the respondents. Surprisingly, 49.4% preferred a pH range between 7.30 and 7.40. Mild hypoxemia with a target SpO_2_ of 88–92% was tolerated by 62.4% of respondents, while 5.9% desired a target SpO_2_ of >95%. In the case of persistent hypoxemia with low PEEP (case scenario 5), 74.9% of respondents considered increasing PEEP, whereas 21.6% switched to high-frequency oscillatory ventilation. Almost all respondents reported performing the lung recruitment maneuvers on patients with moderate to severe pARDS. Three-quarters of ICU physicians preferred decremental PEEP titration, while half of the non-ICU physicians preferred incremental PEEP titration.

### Comparing Results From ICU Physicians to Non-ICU Physicians

**Figure 2:**
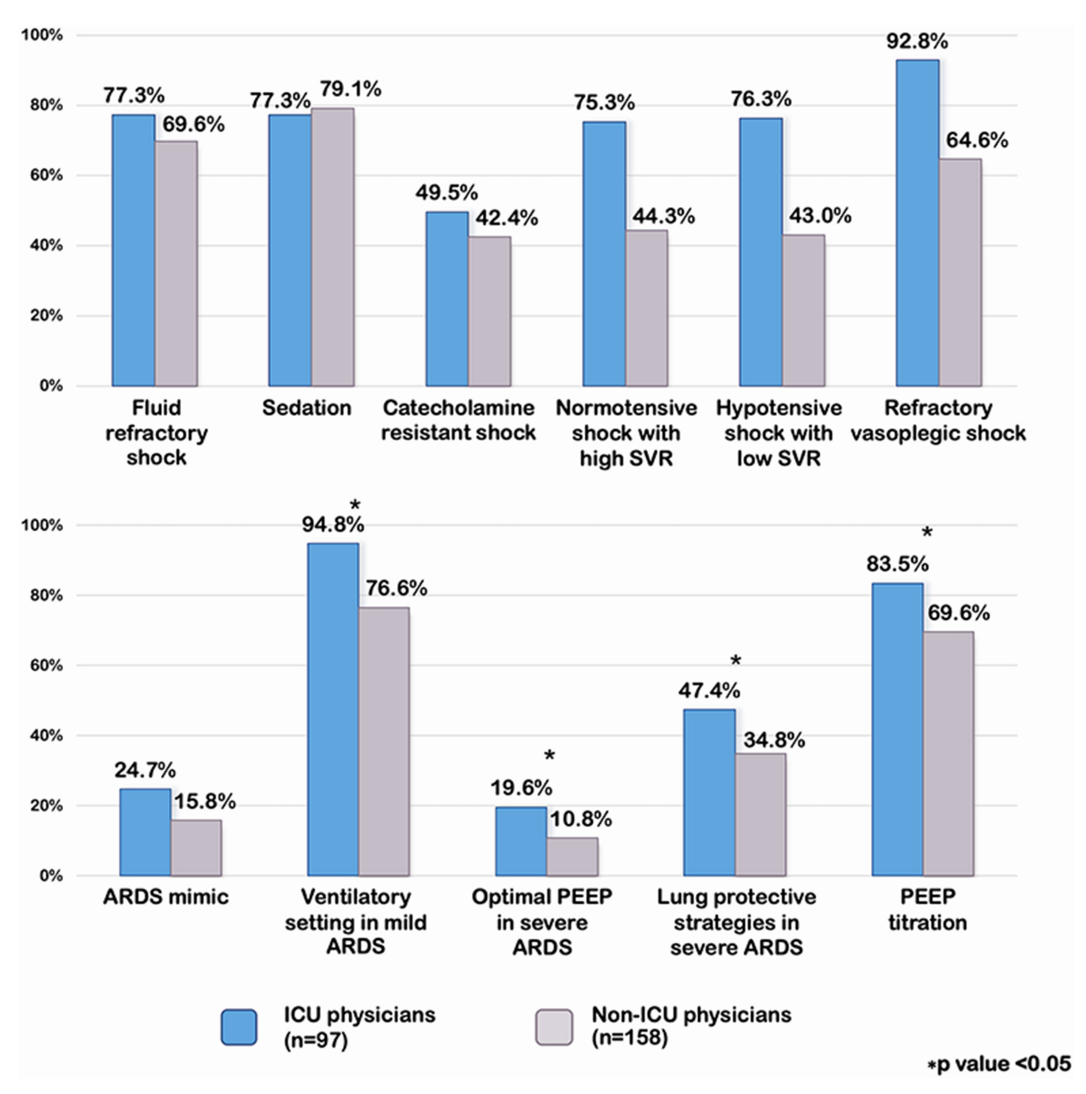
Percentage of appropriate answers on septic shock and pARDS compared ICU and non-ICU physicians. SVR, systemic vascular resistance; PEEP, positive end expiratory pressure; ARDS, acute respiratory distress syndrome.

We analyzed the percentage of an appropriate answers in each scenario comparing ICU and non-ICU physicians. ICU physicians had a significantly higher percentage of an appropriate answers in normotensive shock with high SVR, hypotensive shock with low SVR, and in refractory vasoplegic shock than non-ICU physicians (75.3 vs. 44.3%, *p* < 0.001, 76.3 vs. 43%, *p* < 0.001, and 92.8 vs. 64.6%, *p* < 0.001, respectively) [Figure 2]. However, when a subgroup of 97 ICU physicians were analyzed, the intensivists were more likely to have the appropriate answers than the cardiologists and the pulmonologists (100 vs. 89.5 vs. 88.9% *p* =0.13, respectively). ICU physicians demonstrated significantly greater comprehension of optimal PEEP in severe ARDS and PEEP titration than non-ICU physicians (19.6 vs. 10.8%, *p* 0.05 and 83.5 vs. 69.6%, *p* 0.01, respectively) [Figure 2].

## Discussion

Our study demonstrated a significant level of heterogeneity in the clinical practices among Thai pediatricians, as well as some discrepancies with ACCM and PALLIC guidelines. The choice of first-line inotrope or vasopressor for warm shock was unclear until the 2014 update version of the ACCM, which favored the use of norepinephrine in warm shock (9). According to our survey, the majority of Thai pediatricians chose norepinephrine as a vasopressor of choice, followed by 11.4% who preferred epinephrine. These results corresponded with the previous survey published in 2019 (14), which demonstrated that 60% of pediatric intensivists preferred norepinephrine and 25% chose epinephrine.

The current pediatric sepsis guideline highlighted the hemodynamic effects of sedative and analgesic drugs in vulnerable patients with shock. The preemptive use of ketamine and atropine is considered the best regimen to promote cardiovascular integrity by augmenting SVR and protects against bradycardia (15, 16). Even though 71.8% of hospitals in our survey had ketamine available, only one-fourth of Thai pediatricians use this combination. The fact that general pediatricians are unfamiliar with the use of ketamine may have contributed to this finding.

The role of corticosteroids in catecholamine-resistant shock has been widely debated in both the adult and pediatric literature. Adjunctive corticosteroid hastened the resolution of shock but only demonstrated controversial evidence regarding mortality benefits (17–19). ACCM recommended hydrocortisone therapy in shock despite epinephrine or norepinephrine infusion without clear definition (9). Consequently, physicians providing care are left to make individual decisions at the bedside, resulting in a significant practice variation. Our survey showed that 45.1% of the respondents prescribed hydrocortisone in patients with fluid refractory shock who required one high dose of the vasoactive agent. This was consistent with a previous survey which reported that 50% of physicians prescribed hydrocortisone for patients requiring one high dose vasoactive agent and 91.4% of physicians would prescribe hydrocortisone for patients requiring two or more vasoactive agents (20).

Case scenarios in more complex shock types were created to measure respondents' interpretation and implementation of advanced non-invasive monitoring to patient management. ICU physicians showed more consistent adherence to the guidelines than the non-ICU physicians since management beyond catecholamine-resistant shock requires advanced hemodynamic monitoring and medications. Resource-limited hospitals and unacquaintance to more complex shock for non-ICU physicians might restrict their management practices. In pARDS, we found that Thai pediatricians have quite good adherence to low tidal volumes ventilation with only 1.2% reported using high tidal volumes (>10 mL/kg). These results corresponded with the previous self-reported surveys in North America and Europe, which showed that most of the pediatric intensivists used tidal volumes between 5 and 8 mL/kg, and none of them reported using high tidal volumes (>10 mL/kg) (21). However, they differed from the actual practices in a cross-sectional observational study (PALIVE) taking place in the same population, which reported that ~25% of patients were ventilated with exhaled tidal volumes of >10 mL/kg (13). This highlighted the gap between theoretical knowledge and routine practices. Adequate positive end-expiratory pressure (PEEP) is essential to prevent repetitive opening and closing of the alveoli during the respiratory cycle, which may lead to further ventilator-induced lung injury and is associated with lower mortality. Observational studies in both adults and children showed that many patients with ARDS received lower PEEP than the recommendation (22, 23). We discovered similar results, with just 14.1% of severe ARDS patients receiving optimal PEEP, most of the respondents reported not to use PEEP above 10 cmH_2_O. A retrospective study in 1,134 patients with pARDS illustrated that 26.6% of patients were managed with lower PEEP relative to the amount of FIO_2_ recommended by the ARDSNet protocol. Patients managed with lower PEEP significantly experienced higher mortality than those who were managed with PEEP levels in line with or higher than recommended by the protocol (23). Pediatricians were hesitant to increase PEEP in response to hypoxemia, preferring to increase FiO_2_ instead (13, 23). The reasons were likely multifactorial and might be related to concerns about high PEEP levels in infants and neonates with low chest wall elastance, concerns about cardiopulmonary interactions, or a perception that high FiO_2_ is not harmful (23–25).

A recruitment maneuver is a sustained increase in airway pressure to open collapsed alveoli, followed by sufficient PEEP to keep the lungs open (26). PALLIC guideline recommended careful recruitment maneuvers in the attempt to improve severe oxygenation failure (10). A variety of approaches have been used, including decremental PEEP titration, incremental PEEP titration, sustained inflation with CPAP, intermittent sigh breaths, and others. However, evidence is lacking that one approach is superior to the others, and the choice is determined by individual practice (27, 28). Our study discovered that ICU physicians favored decremental PEEP titration, whereas non-ICU physicians preferred incremental PEEP titration, which might be attributed to the gradual rise of pressure is better tolerated from a hemodynamic standpoint for non-ICU physicians.

Permissive hypercapnia is a ventilation strategy that allows an unphysiologically high partial pressure of carbon dioxide (PCO_2_) to permit lung-protective ventilation with low tidal volumes. Nearly half of the respondents aimed for relatively normal arterial blood gas, highlighting Thai pediatricians' misconceptions about permissive hypercapnia and mild hypoxemia.

Our study had some strengths. Opportunities for critical care training in resource-limited setting are scarce. Our country is a developing country with a scarcity of specialty physicians, infrastructure, and medical equipment. We conducted the first survey of Thai pediatricians regarding their current practices and understanding related pediatric septic shock and pARDS. Our country is a developing country with a scarcity of specialty physicians, infrastructure, and medical equipment. This survey gathered replies from individuals with a variety of professional titles, years of experience, and hospital kinds, ranging from general hospitals to medical schools. In 2018, Thailand adopted a clinical practice guideline for pediatric sepsis and septic shock. Our findings indicated that the majority of participants demonstrated enough knowledge regarding sepsis management. On the other hand, the majority of participants provided an inadequate answer to criticism about pARDS and sophisticated pediatric septic shock management. The survey's findings imply that the local guideline may help improve management adherence. A previous study revealed that critical care is frequently regarded inappropriate and of minor importance than primary care efforts, particularly in resource-limited settings (29). This study may be the first step toward gaining a better understanding of the knowledge, self-reported practice, and skills of local pediatricians caring for children with sepsis and ARDS. Although following international guidelines can improve patient outcomes, there will be some knowledge gaps among pediatricians in developing countries. These knowledge gaps could be reduced by increasing the hands-on workshops and frequently updated conference meetings. In addition, local guidelines for sepsis and pARDS management for non-ICU physicians should be developed, and pediatric critical care fellowship training programs should be promoted as part of national policy to improve quality of care.

Our study may have some limitations. First, it was unclear overall target population since we did not know the exact total number of pediatricians who have been practicing in PICU. Although our study collected from 255 pediatricians, these participants cannot be considered definitively representative of all nations. It was unclear overall target population and an inability to quantify response rates owing to the survey's distribution *via* social media. Our survey, on the other hand, was distributed to all tertiary and university-based hospitals with a pediatric intensive care unit. Second, there was high proportion of pediatricians who was working in the upper level of tertiary center which might limit generalizability. There was also the possibility of selection bias, since pediatricians interested in critical care medicine may be more likely to respond to our questionnaire. Nevertheless, this study could explain the actual practice and perception to manage pediatric septic shock and pARDS patients. Last, the management in a self-reported survey may not accurately reflect real-life practices at the bedside, despite our best efforts to construct scenarios to best suit actual practices.

## Conclusion

This survey added more confirmation on the variability of current self-reported pediatric septic shock and pARDS management practices, as well as knowledge gaps and lack of adherence to guidelines. The variation might be due to resource constraints, unacquaintance to critically ill children, lower grading of pediatric evidence compared to adults, and unclear recommendations of current guidelines. Caring for critically ill children had been increasingly difficult in recent years, highlighting the necessity of pediatric critical care physicians in treating these patients. We emphasized the need for continuous education and training in pediatric intensive care medicine in order to improve the quality of care.

## Data Availability Statement

The original contributions presented in the study are included in the article/supplementary material, further inquiries can be directed to the corresponding author.

## Ethics Statement

The studies involving human participants were reviewed and approved by Human Research Ethics Committee, Faculty of Medicine Ramathibodi Hospital, Mahidol University. The patients/participants provided their written informed consent to participate in this study.

## Author Contributions

PP and NA: contributed to the design of the study, data collection, data analysis, and manuscript drafting. JV, RL, and MC: contributed to the design of the study. PP, NA, and CC: contributed to the initial draft of the manuscript. NA: critically revised it for important intellectual content. All authors gave final approval of the version to be published.

## Conflict of Interest

The authors declare that the research was conducted in the absence of any commercial or financial relationships that could be construed as a potential conflict of interest.

## Publisher's Note

All claims expressed in this article are solely those of the authors and do not necessarily represent those of their affiliated organizations, or those of the publisher, the editors and the reviewers. Any product that may be evaluated in this article, or claim that may be made by its manufacturer, is not guaranteed or endorsed by the publisher.
